# THE PROMISE AND CHALLENGES OF GERONTOLOGISTS WHO ADVANCE THE SCIENCE OF COMMUNITY-ENGAGED RESEARCH

**DOI:** 10.1093/geroni/igad104.1907

**Published:** 2023-12-21

**Authors:** Carrie Leach, Daniel Gan, Allyson Brothers, Lana Sargent, Sarah Dys

**Affiliations:** Wayne State University, Detroit, Michigan, United States; Thompson Rivers University, Kamloops, British Columbia, Canada; Colorado State University, Fort Collins, Colorado, United States; VCU School of Nursing, Ashland, Virginia, United States; Portland State University, Portland, Oregon, United States

## Abstract

Our aging population demands more responsive policies and services and calls for a shift from involving older adults as individual participants toward collaborating with or empowering their communities as research partners. Knowing how to build capacity for community-engaged research (CEnR) among gerontologists requires understanding the challenges of such endeavors. A survey was distributed to gerontologists in March 2021 to December 2022, and was supplemented by a brainstorming focus group. We received responses from academic faculty, staff, students, and trainees (n=98) representing multiple academic disciplines from community health and implementation science to behavioral and social sciences. Many identified as a minoritized person (57.7%) and used mixed methods (55.1%). Focus group findings revealed challenges around community and participant recruitment, sustaining relationships, low-resource contexts and recognition. These reflected concerns around time, work, and funding – three most commonly-occurring words in survey responses. Barriers articulated include, “the time it takes to cultivate real relationships with community partners to design and carry out research together,” “confusion among academic colleagues on how this work is scholarship,” and a “lack of understanding and recognition from universities and funding agencies.” To more effectively mobilize knowledge and remain relevant, institutions would do well to heed calls for training on involving community members as co-researchers (78.6%), learning about rapidly developing CEnR methodologies (75.3%), and sustaining successful intervention programs after grant funding has ended (70.4%) among other priorities. This study provides critical insight into the landscape of CEnR among gerontologists and may be used to develop more supportive programs and structures.

